# Cryopreservation effects on a viable sperm sterlet (*Acipenser ruthenus*) subpopulation obtained by a Percoll density gradient method

**DOI:** 10.1371/journal.pone.0202514

**Published:** 2018-08-16

**Authors:** Yevhen Horokhovatskyi, Mariola A. Dietrich, Ievgen Lebeda, Pavlo Fedorov, Marek Rodina, Borys Dzyuba

**Affiliations:** 1 Research Institute of Fish Culture and Hydrobiology, Faculty of Fisheries and Protection of Waters, University of South Bohemia in Ceske Budejovice, Vodnany, Czech Republic; 2 Department of Gametes and Embryo Biology, Institute of Animal Reproduction and Food Research, Polish Academy of Sciences, Olsztyn, Poland; Fred Hutchinson Cancer Research Center, UNITED STATES

## Abstract

In many fish species, sperm cryopreservation has deleterious effects and leads to a significant decrease in spermatozoa viability. However, the effect of cryopreservation on sperm cells that survive this process and are still viable is not fully understood. The objective of this study was to compare the viability and proteomes of fresh and cryopreserved sterlet (*Acipenser ruthenus*) sperm samples before and after live-dead cell separation using Percoll density gradient centrifugation. Both fresh and cryopreserved sperm samples were divided into two groups (with or without application of Percoll separation). At each step of the experiment, sperm quality was evaluated by video microscopy combined with integrated computer-assisted sperm analysis software and flow cytometry for live-dead sperm viability analysis. Sperm motility and the percentage of live cells were reduced in the cryopreserved group compared to the fresh group from 89% to 33% for percentage of motility and from 96% to 70% for live cells. Straight line velocity and linearity of track were significantly lower in cryopreserved samples than in those separated by Percoll before and after cryopreservation. However, the percentages of motile and live spermatozoa were higher than 90% in samples subjected to Percoll separation. Proteomic analysis of spermatozoa by two-dimensional differences in-gel electrophoresis coupled with matrix-assisted laser-desorption/ionization time-of-flight/time-of-flight mass spectrometry revealed that 20 protein spot abundances underwent significant changes in cryopreserved samples compared to fresh ones. However, only one protein spot was significantly altered when samples before and after cryopreservation followed by Percoll separation were compared. Thus, the results of this study show that cryopreservation leads to minimal proteomic changes in the spermatozoa population, retaining high motility and viability parameters. The results also suggest that global differences in protein profiles between unselected fresh and cryopreserved samples are mainly due to protein loss or changes in the lethal and sublethal damaged cell subpopulations.

## Introduction

Worldwide demand for sturgeon boneless meat and caviar has resulted in an increased requirement for sturgeon aquaculture [1]. At the same time, fish sperm cryopreservation is considered to be a powerful tool in aquaculture as it affords an opportunity for gamete availability synchronization of sperm and eggs, the rational use of sperm, a decrease in the number of males needed in a hatchery, simplification of broodstock maintenance and gamete transport between fish farms [2]. However, despite all of these advantages, cryopreservation presents challenges, as it may strongly impair sperm function and survival and thus decrease reproductive capacity. Moreover, the mechanisms of spermatozoa cryo-injuries are still under intensive study and are not fully understood [2, 3].

To be successfully cryopreserved and used for artificial reproduction in aquaculture, fish sperm must be of the highest quality before freezing as well as after thawing. However, in many fish species, the cryopreservation procedure has deleterious effects and is associated with a significant decrease in sperm viability [4–6]. During freezing and thawing, spermatozoa are exposed to the influence of numerous physical and chemical factors caused by a high solute concentration during slow freezing or intracellular ice formation during rapid freezing [7]. Moreover, water can recrystallize into larger lethal-sized ice crystals during warming as a result of differences in surface free energy and damage the cell [8].

Cryo-injuries caused by crystallization of internal and external water have deleterious effects on the spermatozoa plasma membrane due to changes in lipid membrane composition, organization and its properties. Moreover, the freeze-thaw process also leads to alterations in DNA, protein integrity and protein-protein interactions [9–11]. The defects in sperm protein, in turn, may have a pernicious effect on sperm motility, fertilization ability, and the early stage after fertilization [11, 12]. As a result of all of these deleterious effects, the membrane-bound proteins and intracellular enzymes, as well as other components of the cell, co-elute from spermatozoa when it is damaged [11].

As a consequence, all damage to which the spermatozoa are subjected during the freezing-thawing process results in the appearance of viable, lethal and sublethal damaged cell subpopulations in a thawed sperm mixture. Usually, such a frozen-thawed sperm mixture is an object of routine cryo-biological studies aimed at evaluating the cryopreservation-generated damage to fish sperm proteins and lipids [13, 14]. However, according to our hypothesis, the presence of lethal and sublethal damaged cell subpopulations in the sperm mixture can obscure the real cryopreservation effects on the subpopulation of spermatozoa that survive the cryopreservation process and retain suitable motility parameters. Furthermore, this subpopulation is the only one involved in fertilization, and the presence of lethal and sublethal damaged spermatozoa can negatively influence this process if a post-thaw mixture is used [15, 16].

In mammals, to obtain a higher number of high-quality spermatozoa even from a poor-quality semen, a variety of different sperm separation techniques are widely used. Foremost among these are swim-up, swim-down, fluorescence cell sorting, electrophoresis, migration-sedimentation, glass wool filtration, magnetic activated cell sorting, and density gradient centrifugation [16–19]. In principle, all of these sperm separation methods imitate the natural selection process *in vitro* in which higher-quality viable sperm are separated from other constituents of the ejaculate by actively moving through the cervical mucus. In the case of artificial insemination, fresh mammalian sperm, as well as cryopreserved sperm, needs to be separated because the presence of low-quality, lethal and sublethal damaged spermatozoa subpopulations may become an obstacle during *in vitro* fertilization [15].

The application of these separation techniques to fish sperm is a completely new approach that presents some challenges. It is known that fish spermatozoa are commonly immotile in seminal fluid and for motility initiation, changes in environment osmolality and ionic composition are required [20]. Thus, to apply separation techniques to fish sperm, the spermatozoa should be kept immotile because the motility period after initiation is not as long as in mammalian species. Additionally, the data from fish sperm separation techniques are very limited. To our knowledge, several attempts have been made using separation techniques for fish sperm. Magnetic-activated cell sorting was first applied to Senegalese sole (*Solea senegalensis*) sperm to eliminate the apoptotic spermatozoa subpopulation before cryopreservation [21]. This technique obtained a non-apoptotic sperm subpopulation from low-quality samples and improved artificial fertilization and cryopreservation outcomes. Another separation technique was used for common carp (*Cyprinus carpio*) sperm after a conventional freeze-thaw procedure [22]. The Percoll density gradient centrifugation technique was effective for the removal of most dead spermatozoa and spermatozoa with damaged membranes exposed to the effects of cryopreservation. Therefore, separation of fish spermatozoa has great potential to be applied for the improvement of assisted reproduction in aquaculture; however, the technique requires refinement, specifically depending on the fish species.

In our study, the Percoll density gradient separation technique was used for the first time in sterlet (*Acipenser Ruthenus*) sperm for the selection of a viable spermatozoa subpopulation from a frozen-thawed sperm suspension. The objective of our study was to compare the sperm motility, viability and proteomes of fresh and cryopreserved sterlet sperm before and after cell subpopulation selection using Percoll density gradient centrifugation.

## Materials and methods

The Percoll density gradient centrifugation technique has never been used on sterlet (*Acipenser ruthenus*) sperm. Therefore, this procedure was elaborated for the first time through experimental trial and error. To use the Percoll density gradient centrifugation technique on sterlet sperm, the concentration of the immobilizing saline solution, which keeps the spermatozoa immotile during the entire process of separation, the Percoll concentration in column layers, and centrifugation force and time were determined. The successful application of this technique showing constant and repeated results was obtained on samples from more than 30 individual sterlet males.

### Ethics statement

The methodological protocol of the current study was approved by the expert committee of the Institutional Animal Care and Use Committee (IACUC) of the University of South Bohemia (USB) in Ceske Budejovice, Faculty of Fisheries and Protection of Waters (FFPW) in Vodnany according to the law for the protection of animals against cruelty (Act no. 246/1992 Coll., ref. number 16OZ22302/2014-17214).

### Sample collection

Sterlet (*Acipenser ruthenus*) was selected as the model sturgeon species in this study. All manipulations with sterlet individuals were described in a protocol approved by the committee mentioned above. According to this protocol, sturgeon individuals weighing less than 40 kg are not anaesthetized [23], as the anesthesia leads to a fish recovery time and can provoke sperm loss together with fish suffering from the anesthesia itself. Sample collections were performed in March at the genetic fisheries center of the Faculty of Fisheries and Protection of Waters, Vodnany. Thirty sterlet males (3–4 years old, 0.8–1.6 kg body weight) were transferred from fish farming ponds with a water temperature of 8‒10 °C to 4 m^3^ plastic tanks with a closed water recirculating system. The water temperature in the plastic tanks was increased to 15 °C by a 1 °C increment per day. Spermiation was induced by a single intramuscular injection of carp pituitary powder dissolved in a 0.9% (w/v) NaCl solution at a dose of 4 mg/kg body weight. Twenty-four hours post-stimulation, sperm was collected from each of thirty males using a syringe with an attached 4 mm plastic catheter inserted into the urogenital ducts. Before the start of the experiment, the collected sperm was stored in a flat-bottom container on ice at 4 °C [24].

### Cryopreservation procedure

Freshly collected sperm samples from thirty fish males were diluted at a 1:1 ratio (v: v) using an extender with a buffer consisting of 30 mM Tris, 23.4 mM sucrose, and 0.25 mM KCl supplemented with 10% methanol [4]. After a 10 min equilibration period, 0.5 mL plastic straws were filled with a sperm-extender suspension and placed on a 3 cm thick polystyrene raft. Each set of 20 straws on the raft was then placed in a Styrofoam box (dimensions: 52x33x30 cm) that was filled to a depth of 10 cm with liquid nitrogen. Ten min after exposure to liquid nitrogen vapor (-160 °C), the straws were plunged directly into liquid nitrogen (-196 °C) and stored in plastic goblets prior to thawing. During thawing, samples were exposed to a 40 °C water bath for 6 s [24] followed by immediate use for further estimations and sample processing.

### Sperm separation by Percoll density gradient centrifugation

To obtain the 90% and 40% Percoll solutions, stock Percoll (catalog number P1644, Sigma-Aldrich, USA) was diluted with a 100 × concentrated artificial seminal fluid to yield a final concentration of 16 mM NaCl, 3 mM KCl, 0.19 mM CaCl_2_, 10 mM Tris-HCl buffer at pH 8.0 that is similar to native sterlet seminal fluid [25]. The Percoll density column was set up in a 15 mL plastic tube by smoothly layering 2 mL of 40% Percoll solution on top of 2 mL of 90% Percoll solution. On the top of the column, 1 mL of either fresh or frozen-thawed sperm mixture was gently layered, and then the tubes were centrifuged for 20 min at 2000 × g, 4 °C. A visual presentation of the results of Percoll gradient centrifugation applied to sterlet sperm is shown in S1 Fig. The resulting pellets were washed at a 1:10 ratio with artificial seminal fluid and centrifuged at 3000 × g for 10 min at 4 °C. After resuspension, one part of each pellet underwent sperm parameter evaluation and a live/dead sperm cells ratio estimation, while the other part was frozen at -80 °C prior to proteome analysis. The information regarding sperm concentration of fresh and cryopreserved samples before and after Percoll separation is presented in S1 Table.

### Sperm motility parameters

For sperm motility activation, fresh, fresh-separated, cryopreserved and cryopreserved-separated sperm samples from thirty fish males were diluted at 1:50 in a medium containing 1 mM CaCl_2_, 10 mM Tris-HCl (pH 8.0), and 0.25% Pluronic F-127 (catalog number P2443, Sigma-Aldrich, USA) under a phase contrast microscope (UB 200i, PROISER, Spain). Immediately after activation, the motility was recorded in duplicate with an ISAS 782M digital camera (PROISER, Spain) in AVI format. The resulting video records were analyzed using an Integrated System for Semen Analysis (ISAS) software (PROISER, Spain). Sperm motility evaluations included several parameters: 1.) percentage of motile sperm cells (%MOT); 2.) curvilinear velocity over the actual path in μm/s (VCL); 3.) straight line velocity in μm/s (VSL); and 4.) linearity of track, VSL/VCL * 100% (LIN). All computer-assisted sperm analysis (CASA) parameters were obtained at 10 s intervals from 10 to 90 s post-activation. To describe the changes in VCL, VSL, and LIN over the post-activation time, these parameters were analyzed using linear regression. In this approach, the intercept with the Y-axis can be viewed as a criterion describing the initial value of the sperm motility parameters while the slope can be considered as a value describing the decrease in these parameters over the post-activation time.

### Sperm viability analysis

The live/dead sperm cell ratio was determined for all sperm samples (n = 30) by flow-cytometric analysis using the membrane-permeant SYBR-14 for live cell nucleic acid staining in combination with propidium iodide (PI) for dead cell nucleic acid staining that penetrates through damaged plasma membrane. Prior to measurements, live untreated sperm (negative control) and sperm subjected to repeated freezing-thawing (positive control) were used to calibrate the sensitivity of each fluorescent channel, thresholds, and set-up regions of interest. Artificial seminal fluid samples diluted to a concentration of 10,000 spermatozoa/mL were at first incubated with 5 μL of 20 × diluted from initial concentration SYBR-14 dye (initial concentration: 10,000 × in DMSO, catalog number S9430, Sigma-Aldrich, USA) at 4 °C for 5 min. Thereafter, 5 μL of 4.8 mM propidium iodide was added to the same suspension and incubated for an additional 30 minutes at 4 °C [26]. A minimum of 3,000 sperm cells was analyzed using a CUBE 8 (Partec, Germany) flow cytometer at flow speed 0.2 μL/s. The data were processed using CyView 1.3 (Partec, Germany) software. For each sample, the populations with different intensities in the PI channel were compared and correlated with membrane damage; thus, populations with high PI fluorescent signals were considered dead cells. Based on the ratio between populations with low and high PI fluorescence intensity, the percentage of live and dead sperm cells was calculated.

### Sperm sample preparation for proteomic analysis

Proteins were extracted from fresh and cryopreserved sperm from three sterlet males with or without Percoll separation (12 samples in total). The resulting sperm pellets were thawed at room temperature (23 °C), resuspended in a protein extraction buffer, sonicated on ice and centrifuged in a 4 °C cooled centrifuge. The obtained protein lysates containing approximately 500 μg of sperm proteins were processed with the use of a Clean-Up Kit (GE Healthcare, Sweden) according to the manufacturer’s protocol. All samples were resuspended in lysis buffer to a protein concentration of 5 mg/mL. The protein concentration before and after the cleaning procedure was measured by a Coomassie (Bradford) Protein Assay Kit (Thermo Scientific, Waltham, USA) [27].

### Fluorescent labelling of sample groups with CyDyes and two-dimensional difference gel electrophoresis (2D-DIGE)

Protein labelling with CyDye DIGE fluors (Cy2, Cy3, Cy5) and two-dimensional electrophoresis were performed using the same parameters as described by Dietrich et al. [28]. The Cy2 dye labelled the internal standard while Cy3 and Cy5 dyes were used to label the experimental sample groups, with three replicates per group. An internal standard was obtained by mixing equal parts of protein from each group. On each electrophoresis gel, extracts from two of the various groups after different treatments (Cy3 and Cy5) and internal standard (Cy2) were separated. The labelling reaction was performed in the dark on ice for 30 min. Differently labelled samples were mixed together according to the scheme presented in Table 1. Passive rehydration was performed using a rehydration buffer that was added to each sample mixture to reach a final volume of 350 μL and loaded onto immobilized pH gradient strips (IPG, 24 cm, pH 3–10 NL). Isoelectric focusing (IEF) was performed with an Ettan IPGphor device (GE Healthcare) as described by Dietrich et al. [28]. After IEF, the strips were equilibrated with an SDS equilibration buffer containing 10 mg/mL DTT for 15 min in the first step and in the second step with SDS equilibration buffer containing 25 mg/mL iodoacetamide for another 15 min.

**Table 1:** Mixing and Cy-dying scheme of fresh and cryopreserved samples with or without application of Percoll separation; n = 3 for each group.

Gel no.	Cy2 (50 μg)	Cy3 (50 μg)	Cy5 (50 μg)
1	Pooled standard	Fresh 1	Fresh-separated 3
2	Pooled standard	Fresh-separated 1	Cryopreserved-separated 2
3	Pooled standard	Cryopreserved 1	Fresh 3
4	Pooled standard	Cryopreserved -separated 1	Cryopreserved 3
5	Pooled standard	Fresh 2	Cryopreserved -separated 3
6	Pooled standard	Fresh-separated 2	Cryopreserved 2

As a part of 2D gel electrophoresis, the equilibrated strips were then transferred to a 12.5% SDS-PAGE (gel size 25.5 × 19.6 cm, 1 mm thickness; GE Healthcare) and sealed with 0.5% agarose. The gel was run at 1.5 W/gel in an Ettan Dalt-Six device (GE Healthcare) for 16 h [28]. Once the bromophenol blue reached the anode, electrophoresis was completed.

### Image acquisition and analysis of two-dimensional gels

After electrophoresis, the stained 2D gels were scanned on a Typhoon 9500 FLA scanner (GE Healthcare) using the parameters recommended by the manufacturer for 2D-DIGE experiments. Image analysis was performed using the DeCyder Differential In-Gel Analysis version 5.02 software (GE Healthcare) in order to identify fluorescent areas. The DeCyder biological variation analysis module was applied to detect protein spots and concurrently match all twelve protein spot maps from six gels using several parameters: 1.) estimated number of spots at 10,000 and 2.) minimum spot size at 3,000. Only protein spots with a P<0.05 by t-test analysis that showed at least a 1.2-fold increase or decrease in their relative intensities in any comparison between all groups were significantly different. To properly pick and identify the selected spots, DIGE gels were stained using Coomassie Brilliant Blue G-250 (Bio-Rad, Hercules, CA).

### Protein identification using MALDI-TOF/TOF

All spot preparations and identification were performed according to Dietrich et al. [28]. Spots of interest were excised from Coomassie stained two-dimensional gels and washed. After washing, the solution was discarded while spots were dried. Thereafter, samples were incubated overnight in a modified sequencing grade trypsin (Promega, Madison, WI, USA) solution and ammonium bicarbonate. After digestion, the spots were placed in trifluoroacetic acid (TFA) and desalted with Zip-Tip C-18 pipette tips (Millipore, Billerica, MA, USA). Each Zip-Tip was first washed with 100% acetonitrile (ACN) and equilibrated with 50% ACN in 0.1% TFA and 0.1% TFA in water. When Zip-Tip washing and equilibration were finished, the peptides were loaded onto the Zip-tip and then eluted with 2 μL of 50% ACN in 0.1% TFA. After elution, samples were mixed with 2 μL of the matrix solution, and half of this mixture was spotted onto the matrix-assisted laser desorption/ionization target plate (MT 34 Target Plate Ground Steel; Bruker Daltonics) and left to dry.

Matrix-assisted laser-desorption/ionization time-of-flight/time-of-flight mass spectrometry (MALDI-TOF/TOF MS) analysis was performed using a matrix-assisted laser desorption/ionization time-of-flight tandem mass spectrometer (Autoflex Speed TM, Bruker Daltonics). The MS and MS/MS LIFT spectra of selected ions were collected and calibrated externally using monoisotopic protonated ion peptide calibration standards (Bruker Daltonics) and imported to BioTools (Bruker Daltonics). The data were searched using MascotServer (Matrix Sciences) in the INCIr database (2016.04.26) with the specific MASCOT settings: 1.) cleavage enzyme: trypsin, max missed cleavages: 2 and mass tolerance mono: 50 ppm, fragment ion mass tolerance of 0.5 Da; 2.) parent ion mass tolerance of 200 ppm; 3.) alkylation of cysteine by carbamidomethylation as a fixed modification; and 4.) oxidation of methionine as a variable modification. For the PMF and MS/MS ion search, statistically significant (P ≤0.05) matches by MASCOT were regarded as correct hits. Hypothetically identified proteins were searched in terms of protein sequence similarity using the Basic Local Alignment Search Tool (BLAST) [29].

### Ingenuity pathway analysis of differentially abundant proteins

An Ingenuity Pathway Analysis (IPA, Ingenuity, Mountain View, CA) was performed to uncover the significance of potential biological pathways. As IPA only accepts gene or protein accession numbers representing human, mouse, and rat genes or proteins, orthologs of the identified sturgeon proteins belonging to those three species were first identified and the accession numbers of the top blast hits were uploaded to IPA. Each identifier was associated with the IPA knowledge base and used to perform functional and canonical pathway analysis. Fisher's exact test and Benjamini–Hochberg multiple testing corrections were used to calculate the significance (P < 0.05) of functional and canonical pathways of proteins changed upon cryopreservation.

### Protein-protein interaction analysis

A protein-protein interaction analysis was performed using a web-based bioinformatic Search Tool for the Retrieval of Interacting Genes/Proteins (STRING) software (https://www.string-db.org/), version 10.5. The identified sperm proteins that underwent significant changes in their abundance after cryopreservation were entered into STRING for analysis of potential protein-protein interactions. The search of interactions was restricted to *Homo sapiens* (Human, 9606) protein pairs. The reliability of the interactions between proteins was assessed by a combined score (edge score).

### Statistical analysis

The experiments evaluating sperm motility, live/dead sperm cells ratio and the Percoll separation success were conducted with sperm from thirty sterlet males. The values of the percent of motile spermatozoa and live/dead cells were expressed as the mean ± standard deviation (±SD). Due to the low number of samples, a nonparametric Kruskal-Wallis test followed by the Mann-Whitney U-test with Bonferroni correction was applied to determine differences between groups. The criterion for significance was P < 0.05 [30]. VCL, VSL, and LIN parameters obtained from the motility measurement from more than 10,000 spermatozoa were subjected to an estimation of mean and standard deviation at 10‒90 s (in 10 sec increments) post-activation times. The resulting mean and standard deviations values were used to plot linear regression lines and determination of the slope (a), intercept (b), coefficient of determination (R^2^), and P-value for R^2^, whereas testing the hypothesis for the equality of regression slopes by ANCOVA were performed using GraphPad Prism version 6 for Windows software (La Jolla, CA, USA). When ANCOVA concluded that the slopes were not all equal, the t-test with Bonferroni correction was applied to detect the significance of difference between coefficients of individual lines (the criterion for significance was P < 0.05) [31]. All statistical significance analyses for sperm motility parameters and live/dead cell percentages were carried out using the Statistica V 12.0 computer program (Statsoft, Inc., Tulsa, OK, USA).

Statistical analysis of changes in protein abundance was performed using the Biological Variance Module of DeCyder Differential In-Gel Analysis version 5.02 software (GE Healthcare) on three biological replicates (individual fishes). The data are expressed as log standardized abundance to ensure a normal distribution of the data. A t-test and average ratio test were performed. Changes in protein spot abundance were considered statistically significant at P < 0.05 with a fold change of 1.2.

Principal component analysis (PCA) was performed in the Extended Data Analysis module of the DeCyder software to visualize the differences between fresh, fresh-separated, cryopreserved and cryopreserved-separated sperm groups. PCA creates and coordinates the points representing samples by using the total log standardized abundance of spots on a specific gel. This analysis emphasizes the variation and brings out strong data patterns obtained from protein spots in the form of principal components in which the first component explains the largest proportion of the variance.

## Results

### Sperm motility parameters and viability before and after cryopreservation

**Fig 1:**
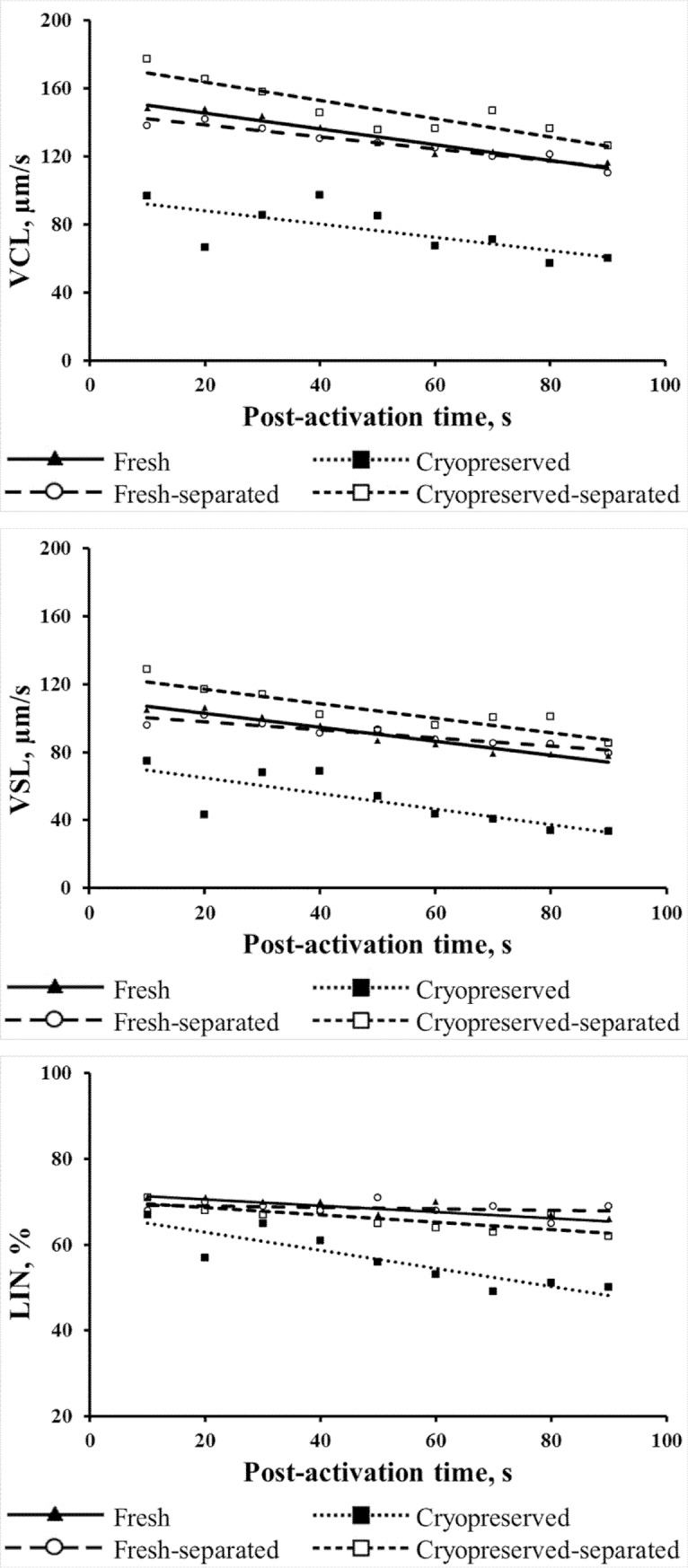
Linear regression lines describing changes in sperm motility parameters over the post-activation time. VCL = curvilinear velocity over the actual path, in μm/s; VSL = straight line velocity in μm/s; LIN = linearity of track, VSL/VCL * 100%. The lines are plotted on the values of the mean ± SD.

The cryopreservation procedure led to a significant decrease in sperm motility percentage in frozen-thawed samples compared to fresh ones (Table 2). The highest percentage of motile sperm cells was observed in samples that underwent the Percoll separation procedure. The value increased from 89.3% to 97.7% for separated fresh sperm, while the value of separated samples after cryopreservation samples significantly increased from 33.8% to 96.2%. The Percoll separation caused a significant increase in the sperm motility percentage (Table 2) and motility parameters (Fig 1). The significantly lowest value of live sperm cells was observed in cryopreserved samples compared to the fresh, fresh-separated and cryopreserved-separated groups. No significant differences were observed between the fresh, fresh-separated and cryopreserved-separated sample groups.

**Table 2:** Sperm motility and viability percentages of thirty sperm samples before and after cryopreservation and Percoll separation.

Parameter	Treatment			
	Fresh	Fresh-separated	Cryopreserved	Cryopreserved-separated
Motility, %	89.3 ± 6.5^a^	97.7 ± 2.3^a^	33.8 ± 11.5^b^	96.2 ± 5.1^a^
Live cell, %	96.8 ± 0.6^a^	98.4 ± 0.5^a^	70.3 ± 2.2^b^	96.8 ± 1.1^a^

^a-b^Means within a row with different superscripts differ (P < 0.05).

The dependencies of VCL, VSL, and LIN over the post-activation time are presented as linear regression lines (Fig 1). The curvilinear velocity (VCL) showed a similar downward slope in all groups (P > 0.05) over the post-activation time, while a sharper decrease (P < 0.05) in track linearity (LIN) was observed in samples after cryopreservation (Table 3). Moreover, the b values showed that the VCL and VSL parameters were initially lower in the cryopreserved samples than in the fresh samples and samples after Percoll separation. As a result of the application of Percoll separation, the highest VCL and VSL parameters were achieved in cryopreserved samples, while the LIN parameter recovered to similar levels in the fresh and Percoll-separated groups.

**Table 3:** The parameters of the linear regression lines describing the sperm motility characteristics obtained with the CASA system.

CASA^1^	Treatment	Parameter^2^			
		R^2^	P for R^2^	a ± SD	b ± SD
VCL	Fresh	0.9450	0.0001	-0.461 ± 0.042^a^	154.6 ± 2.4^a^
	Fresh-separated	0.9265	0.0001	-0.353 ± 0.038^a^	145.6 ± 2.1^b^
	Cryopreserved	0.8557	0.0041	-0.538 ± 0.125^a^	95.7 ± 9.1^c^
	Cryopreserved-separated	0.7749	0.0017	-0.536 ± 0.109^a^	174.0 ± 6.1^d^
VSL	Fresh	0.9399	0.0001	-0.407 ± 0.039^a^	111.0 ± 2.2^a^
	Fresh-separated	0.8705	0.0002	-0.238 ± 0.035^b^	102.7 ± 1.9^b^
	Cryopreserved	0.8764	0.0006	-0.611 ± 0.094^c^	84.2 ± 5.5^c^
	Cryopreserved-separated	0.7475	0.0026	-0.426 ± 0.094^a^	125.5 ± 5.3^d^
LIN	Fresh	0.7439	0.0044	-0.073 ± 0.021^a^	72.1 ± 1.2^a^
	Fresh-separated	0.6980	0.0051	-0.052 ± 0.017^a^	71.1 ± 1.1^a^
	Cryopreserved	0.9431	0.0001	-0.317 ± 0.029^b^	70.2 ± 1.7^a^
	Cryopreserved-separated	0.6681	0.0071	-0.085 ± 0.023^a^	70.4 ± 1.3^a^

^a-c^Means within a column with different superscripts are different (P < 0.05).

^1^CASA: VCL = curvilinear velocity over the actual path, in μm/s; VSL = straight line velocity in μm/s; LIN = linearity of track, VSL/VCL * 100%.

^2^Parameters: R^2^ = coefficient of determination; a = slope, which can be considered as a value describing the decrease of sperm motility parameters over the post-activation time; b = intercept, initial value of the sperm motility parameters.

### Protein expression in 2D-DIGE and identification of different protein spots by matrix-assisted laser desorption/ionization time-of-flight/time-of-flight mass spectrometry (MALDI-TOF/TOF)

**Fig 2:**
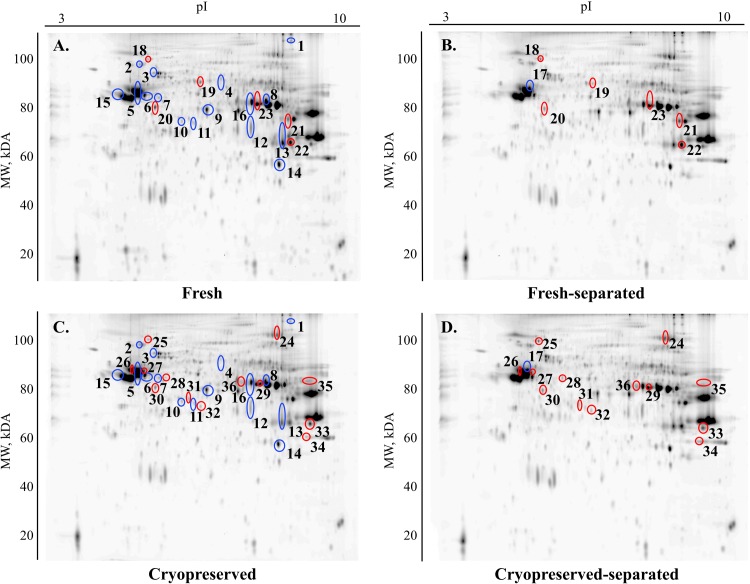
Representative 2D differences in-gel electrophoresis gels showing the comparison of proteomic profiles of the groups. The protein spots identified because they underwent significant changes in abundance are marked with different colors: 1.) fresh compared to cryopreserved sperm groups (blue color A, C); 2.) fresh-separated compared to cryopreserved-separated sperm groups (blue color B, D); 3.) fresh compared to fresh separated sperm groups (red color A, B); and 4.) cryopreserved compared to cryopreserved-separated sperm groups (red color C, D). pI = isoelectric point; MW = molecular weight.

**Fig 3:**
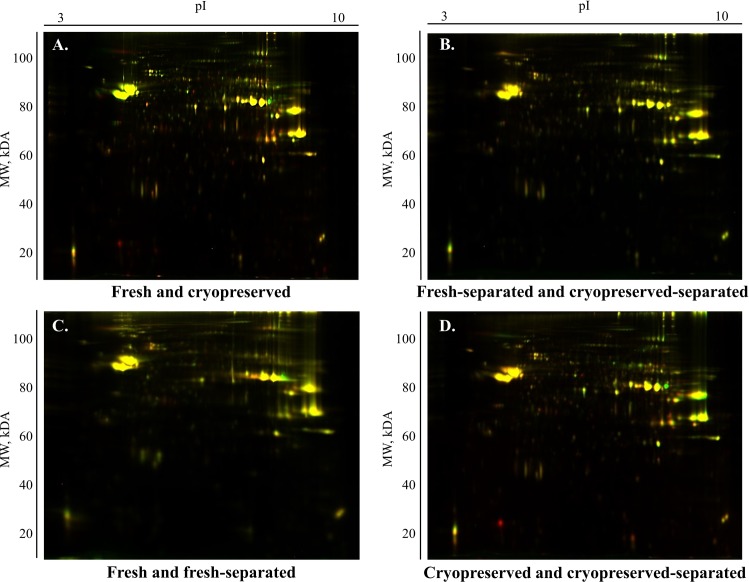
Representative 2D differences in-gel electrophoresis gels. The overlay of fresh (Cy5 dye, red) and cryopreserved (Cy3 dye, green) sperm groups (A), fresh-separated (Cy3 dye, green), and cryopreserved-separated (Cy5 dye, red) sperm groups (B), fresh (Cy3 dye, green) and fresh separated (Cy5 dye, red) sperm groups (C), cryopreserved (Cy5 dye, red), and cryopreserved-separated (Cy3 dye, green) sperm groups (D). Protein spots colored yellow have similar intensities. pI = isoelectric point; MW = molecular weight.

Analysis of the DIGE gels from fresh, fresh-separated cryopreserved and cryopreserved-separated sperm sample groups revealed 1566 matched spots; among these, 60 spots had significant differences (P < 0.05). A quantitative comparison of the proteome profiles of fresh and cryopreserved sperm samples revealed that 20 protein spots underwent significant changes (P < 0.05) in abundance (Fig 2A and 2C) while differences for only one protein spot were significant when comparing fresh and cryopreserved sperm after Percoll separation (Fig 2B and 2D). The comparison of fresh samples before and after Percoll separation showed significant changes in 11 proteins (Fig 2A and 2B), while 28 proteins underwent significant changes in their contents due to Percoll separation of cryopreserved samples (Fig 2C and 2D). A representative 2D-DIGE image of an overlay of fresh compared to cryopreserved spermatozoa, fresh compared to cryopreserved sperm after Percoll separation, fresh compared to fresh-separated spermatozoa, and cryopreserved compared to cryopreserved-separated spermatozoa is presented in Fig 3.

MALDI-TOF/TOF analysis allowed the identification of 16 out of 20 selected protein spots (Table 4) when comparing fresh and cryopreserved sperm, while one protein spot was identified after comparison of fresh and cryopreserved sperm after Percoll separation. Out of 11 different protein spots, six were identified when comparing fresh samples before and after separation, and 13 of 28 selected protein spots were identified by comparison of cryopreserved samples before and after separation.

**Table 4:** List of identified sterlet sperm proteins whose abundance changed significantly (P < 0.05; fold change ± 1.2) during cryopreservation or Percoll separation and identified by matrix-assisted laser desorption/ionization (MALDI) time-of-flight/time-of-flight mass spectrometry and fold change of protein abundance between fresh and cryopreserved, fresh-separated and cryopreserved separated, cryopreserved and cryopreserved-separated, fresh and fresh-separated sperm groups.

Spot no.	Protein name	Gene name	GI^a^ number	Organism	Protein score	MW^b^,Da/pI^c^	Number of peptides (ion score ≥ 30)	Sequence coverage, %	Fold change	P-value
Fresh compared to cryopreserved										
1	L-lactate dehydrogenase A chain	LDHA	gi|975115072	*Gekko japonicus*	103	36866/7.71	1	17	-1.25	0.005
2	Histone H3-like	H3F3A	gi|688563016	*Branchiostoma belcheri*	218	59409/10.9	4	16	1.34	0.008
3	heat shock protein 70	HSPA8	gi|302566321	*Acipenser baerii*	355	71158/5.28	3	25	1.25	0.024
4	Serine/threonine-protein kinase SIK2, partial	SIK2	gi|565316864	*Ophiophagus hannah*	80	8509/6.25	1	61	-1.64	0.006
5	tubulin alpha chain, testis-specific	TUBA4A	gi|831271688	*Clupea harengus*	1010	46473/5.01	8	61	1.28	0.027
6	mitochondrial H+-transporting ATP synthase F1 complex beta polypeptide	ATP5A1	gi|296802112	*Rousettus leschenaultii*	750	51384/5.11	7	50	1.39	0.002
7	cytochrome b-c1 complex subunit 1, mitochondrial-like	UQCRC1	gi|591370457	*Chelonia mydas*	210	50681/5.7	2	14	1.38	0.001
8	phosphoglycerate kinase	PGK1	gi|46849425	*Acipenser baerii*	616	42021/5.76	7	44	-1.4	0.004
9	creatine kinase B-type-like isoform X1	CKB	gi|1020521224	*Sinocyclocheilus grahami*	498	43152/5.42	5	27	-1.2	0.044
10	isocitrate dehydrogenase [NAD] subunit alpha, mitochondrial	IDHA1	gi|45361551	*Xenopus tropicalis*	220	40297/6.33	2	22	1.21	0.001
11	isocitrate dehydrogenase 3 (NAD+) alpha	IDH3A	gi|148227952	*Xenopus laevis*	240	40595/6.76	2	14	1.16	0.011
12	glycerol-3-phosphate dehydrogenase [NAD(+)], cytoplasmic	GPD1	gi|663288276	*Calypte anna*	155	34729/6.66	3	16	-1.28	0.020
13	L-lactate dehydrogenase A chain	LDHA	gi|725549051	*Saimiri boliviensis boliviensis*	185	36796/7.08	3	29	-1.23	0.037
14	triose phosphate isomerase	TPI1	gi|46849427	*Acipenser baerii*	437	22890/6.1	5	51	-1.24	0.011
15	tubulin beta-4B chain	TUBB4B	gi|831324610	*Clupea harengus*	1110	50253/4.79	9	56	1.32	0.031
16	phosphoglycerate kinase	PGK1	gi|46849425	*Acipenser baerii*	520	42021/5.76	6	56	-1.26	0.011
Fresh-separated compared to cryopreserved-separated										
17	tubulin alpha-8 chain-like	TUBA8	gi|927153352	*Thamnophis sirtalis*	92	32528/4.48	1	13	1.15	0.042
Fresh compared to fresh-separated										
18	transitional endoplasmic reticulum ATPase	SEC16B	gi|1025248139	*Sinocyclocheilus rhinocerous*	225	89982/5.14	3	15	1.21	0.034
19	keratin type IIE	KRT3	gi|32452105	*Acipenser baerii*	171	51448/5.06	1	28	-2.1	0.005
20	beta-actin	ACTB	gi|119943232	*Misgurnus anguillicaudatus*	730	42038/5.29	7	41	-1.96	0.020
21	creatine kinase U-type, mitochondrial	CKMT1A	gi|38488694	*Danio rerio*	236	47133/8.05	2	15	1.31	0.041
22	L-lactate dehydrogenase A chain	LDHA	gi|573909562	*Lepisosteus oculatus*	216	36934/7.12	2	19	-1.1	0.025
23	phosphoglycerate kinase	PGK	gi|46849425	*Acipenser baerii*	520	42021/5.76	6	56	1.06	0.045
Cryopreserved compared to cryopreserved-separated										
24	glycogen phosphorylase, muscle form	GPH1	gi|946678688	*Pelodiscus sinensis*	408	97539/6.77	6	28	1.4	0.048
25	transitional endoplasmic reticulum ATPase	tER	gi|1025248139	*Sinocyclocheilus rhinocerous*	225	89982/5.14	3	15	1.23	0.003
26	tubulin alpha chain, testis-specific	TUBA	gi|831271688	*Clupea harengus*	1010	46473/5.01	8	61	-1.3	0.040
27	ATP synthase subunit beta, mitochondrial-like	ATP2	gi|432849647	* *	714	55238/5.1	7	47	-1.26	0.002
28	cytochrome b-c1 complex subunit 1, mitochondrial-like	MZM1	gi|591370457	*Chelonia mydas*	210	50681/5.7	2	14	-1.36	0.006
29	phosphoglycerate kinase	PGK1	gi|46849425	*Acipenser baerii*	616	42021/5.76	7	44	1.34	0.019
30	beta-actin	ACTB	gi|119943232	*Misgurnus anguillicaudatus*	730	42038/5.29	7	41	-1.22	0.015
31	isocitrate dehydrogenase [NAD] subunit alpha, mitochondrial	IDP2	gi|45361551	*Xenopus tropicalis*	220	40297/6.33	2	22	-1.3	0.001
32	isocitrate dehydrogenase 3 (NAD+) alpha	IDH3A	gi|148227952	*Xenopus laevis*	240	40595/6.76	2	14	-1.38	0.012
33	voltage-dependent anion-selective channel protein 2 isoform X1	VDAC	gi|1020410464	*Sinocyclocheilus grahami*	672	30351/8.83	5	38	-1.21	0.004
34	phosphoglycerate mutase 2	PGAM2	gi|471409135	*Trichechus manatus latirostris*	304	29037/8.84	3	30	1.68	0.015
35	fructose-bisphosphate aldolase A-2	IOIJ	gi|46849415	*Acipenser baerii*	118	36299/8.1	1	24	1.34	0.029
36	phosphoglycerate kinase	PGK1	gi|46849425	*Acipenser baerii*	393	42021/5.76	5	30	1.22	0.042

^a^GI = GenInfo Identifier.

^b^MW = molecular weight

^c^pI = isoelectric point.

**Fig 4:**
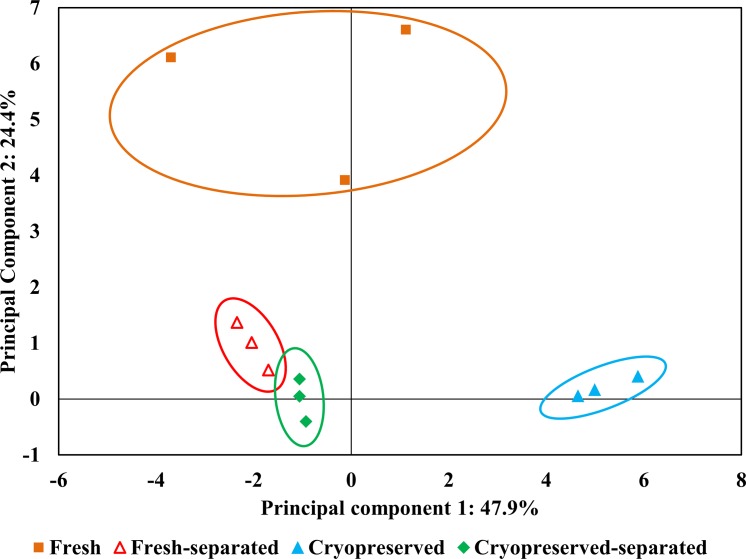
Principal component analysis of abundance data from sturgeon sperm proteins significantly changed during the cryopreservation. Each symbol represents a gel image of one sample. Groups of samples defined by principal component analysis are enclosed in circles.

Principal component analysis (PCA) distinguished differences in the abundance of 43 protein spots in fresh, fresh-separated, cryopreserved, and cryopreserved-separated sperm protein groups (Fig 4). The first principal component accounted for 47.9% of the total variance, suggesting that the greatest differences in protein abundance occurred between cryopreserved semen and the three remaining groups. The highest changes in protein abundance within the groups were between cryopreserved and fresh sperm, then between cryopreserved and fresh-separated sperm, and finally between the cryopreserved and cryopreserved-separated groups. The second principal component accounted for 24.4% of the total variance and showed minimal differences in protein abundance between the fresh and fresh-separated groups and between the fresh and cryopreserved-separated groups. Differences between the fresh-separated and cryopreserved-separated groups were not observed.

### Ingenuity pathway analysis of differentially abundant proteins

The gene name for each identified protein spot that underwent a significant change in abundance due to the cryopreservation process was subjected to IPA analysis to identify its signaling pathway and function (Table 5). These changed sperm proteins were associated with the generation of precursor metabolites and energy, nucleic acid metabolism, and cellular assembly and organization in the top molecular and cellular function category.

**Table 5:** Ingenuity pathway analysis of differentially abundant proteins between fresh and cryopreserved sperm groups.

Top molecular and cellular functions	P-value range	No. of molecule	Protein^a^
Generation of precursor metabolites and energy	2.01e-05–2.75e-07	6	GPD1, TPI1, PGK1, ATP5A1, IDH3A, LDHA
Nucleic acid metabolism	2.21e-05–2.65e-07	5	ATP5A1, GPD1, HSPA8, IDH3A, PGK1
Cellular assembly and organization	2.01e-05–2.75e-07	5	GPD1, TPI1, PGK1, ATP5A1, IDH3A, LDHA
Top canonical pathways	p-Value	No. of molecule	Protein^a^
Sirtuin Signaling Pathway	1.53e-05	4	ATP5A1, H3F3A, PGK1, TUBA4A
Glycolysis	9.34e-05	4	PGK1, TPI1, LDHA, GPD1
Oxidative phosphorylation	1.65e-03	2	ATP5A1, UQCRC1

^a^Proteins: LDHA—l-lactate dehydrogenase A chain; H3F3A - histone H3-like; HSPA8—heat shock protein 70; TUBA4A - tubulin alpha chain, testis-specific; ATP5A1—mitochondrial H+-transporting ATP synthase F1 complex beta polypeptide; UQCRC1—cytochrome b-c1 complex subunit 1, mitochondrial; PGK1—phosphoglycerate kinase; IDH3A - isocitrate dehydrogenase [NAD] subunit alpha, mitochondrial; GPD1—glycerol-3-phosphate dehydrogenase [NAD(+)], cytoplasmic; TPI1—triose phosphate isomerase.

Furthermore, IPA demonstrated that the sirtuin signaling pathway, glycolysis and oxidative phosphorylation were the canonical pathways that were most affected by differentially abundant proteins.

### Protein-protein interaction analysis

**Fig 5:**
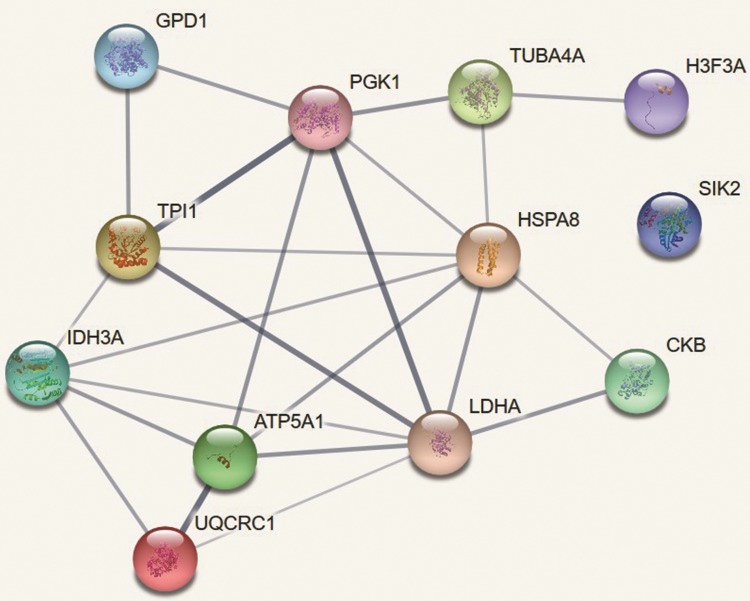
STRING analysis of proteins-protein interactions of differentially abundant proteins. The interaction between fresh and cryopreserved sperm samples is presented. The line thickness indicates the strength of data support. LDHA—l-lactate dehydrogenase A chain; H3F3A - histone H3-like; HSPA8—heat shock protein 70; SIK2 –serine/threonine-protein kinase SIK2; TUBA4A - tubulin alpha chain, testis-specific; ATP5A1—mitochondrial H+-transporting ATP synthase F1 complex beta polypeptide; UQCRC1—cytochrome b-c1 complex subunit 1, mitochondrial; PGK1—phosphoglycerate kinase; CKB—creatine kinase B-type; IDH3A - isocitrate dehydrogenase [NAD] subunit alpha, mitochondrial; GPD1—glycerol-3-phosphate dehydrogenase [NAD(+)], cytoplasmic; TPI1—triose phosphate isomerase; TUBB4B - tubulin beta-4B chain.

The protein-protein interactions network showed medium edge (score 0.4‒0.6) confidence for 43 pairs of interactions and high interactions (score 0.7–0.9) for 10 protein pairs when comparing fresh and cryopreserved sperm groups (Fig 5). The highest interaction (score 0.9) was observed between l-lactate dehydrogenase A and phosphoglycerate kinase 1, mitochondrial H+-transporting ATP synthase F1 complex beta polypeptide and cytochrome b-c1 complex subunit 1, tubulin alpha chain, testis-specific and tubulin beta-4B chain, and phosphoglycerate kinase and triose phosphate isomerase. Such high interactions with high scores indicate functional protein linkages.

## Discussion

In the present study, for the first time, the Percoll density gradient centrifugation method was used for the selection and analyses of spermatozoa possessing high viability and motility in sterlet (*Acipenser ruthenus*) sperm. Moreover, it is the first study that focused on the investigation of changes in protein profiles of the spermatozoa population containing more than 95% of viable cells after the cryopreservation process.

Selection of high-quality, high-density spermatozoa that maintain their fertilization capacity is a crucial factor for the success of assisted reproduction techniques, since dead and abnormal spermatozoa as well as sperm debris may be an obstacle to successful fertilization in *in vitro* conditions [32]. In mammals, a broad range of techniques for sperm separation (sperm selection) is widely used for the improvement of assisted reproduction technologies. Sperm selection techniques mainly separate good-quality spermatozoa, which are usually high density, from the immotile sperm and other constituents of the seminal fluid [33]. This approach significantly improves sperm quality, thereby increasing the amount of progressively motile spermatozoa. For *in vitro* insemination application, both fresh and cryopreserved mammalian sperm should be separated or sorted [16, 34, 35]. The main rationale for such sorting takes into account the fact that the active separation of the most actively swimming spermatozoa that occurs during migration through the cervical mucus is the normal process under *in vivo* conditions [15] but that this process does not occur *in vitro*.

In contrast to these technologies applied to mammalian sperm, the separation of fish sperm is a totally new approach that has never been developed to this extent and presents some challenges. Due to characteristics specific to fish spermatozoa, such as motility activation dependency on environmental osmolality and ionic composition, and the necessity to keep spermatozoa immotile before fertilization [36, 37], the application of these separation techniques can be quite challenging. However, despite such challenges, several attempts have been made in this direction. Previously, Valcarce et al. [21] implemented a sperm selection method for non-apoptotic sperm subpopulation selection in Senegalese sole (*Solea senegalensis*) sperm before cryopreservation. In particular, magnetic-activated cell sorting was used to eliminate the apoptotic spermatozoa subpopulation before cryopreservation. Another example of the application of sperm separation techniques is in common carp (*Cyprinus carpio*) sperm. Li et al. [22], working with cryopreservation of carp spermatozoa, obtained a fraction of higher-quality spermatozoa using Percoll density gradient centrifugation after cryopreservation. They observed a significantly greater proportion of motile spermatozoa with higher velocity and intact membranes in cryopreserved carp sperm after Percoll separation. Similarly, an increased percentage of motile sperm cells with higher motility parameters after Percoll selection were observed in our study. Moreover, the motility percentage of cryopreserved and fresh sperm following the separation by Percoll was higher than the percentage recorded for fresh sperm motility. This indicates that Percoll separation results in the selection of the spermatozoa population presenting the best motility parameters, even in the case of fresh sperm.

A decrease in sperm motility parameters due to the impact of cryopreservation is a well-known phenomenon, and our findings are consistent with those described previously in several fish species [6, 38–41]. Our results show a downward slope in the parameters VCL, VSL and LIN over the post-activation time. Nevertheless, the most substantial decrease in the initial VCL and VSL parameters according to the intercept value was observed for cryopreserved samples. These changes were associated with the presence of a large number of lethal and sublethal damaged spermatozoa that appeared due to the damage engendered by the cryopreservation process and that had an impact on the mean values of these parameters. However, along with an overall decline in the movement characteristics of cryopreserved sperm, the parameters of cryopreserved and Percoll separated sperm showed the highest values of VCL and VSL. A similar phenomenon was observed by Boryshpolets et al. [42], in which the cryopreserved sperm showed higher average velocity parameters than fresh sperm. We suppose that the sperm population possessing lower motility parameters was presented in samples after cryopreservation and then was removed from the post-thaw sperm mixture by Percoll separation.

The application of SYBR-14 and PI fluorescent dyes was successful to determine the live/dead cell ratio in sturgeon sperm samples. A low motility percentage and quite high live cell percentage was observed in the cryopreserved group. These can be attributed to the presence of sperm cells that saved an intact permeability barrier (cell membrane) but that have lost the ability for movement activation. A similar phenomenon was reported by Drobnis et al. [34] in human semen after cryopreservation. Nevertheless, the determination in our study of the percentage of live sperm was one of the parameters showing the efficiency of the Percoll separation technique.

There are many probable causes for sperm cryo-damage. Some authors have reported damage to sperm structure or function during the cryopreservation process due to osmotic stress, oxidative stress or ice crystal formation both within the sperm cells and in the external medium [43, 44]. It was also noted that much damage can occur at the level of the sperm plasma membrane through changes in lipid membrane composition, organization, and properties [45, 46]. However, the freeze-thaw process also leads to alterations in DNA integrity and protein profiles [9, 10, 22]. The defects appearing in sperm proteins may in turn have a pernicious effect on sperm motility and fertilization ability and even on the resulting embryo at the early stages of development following fertilization [11, 12]. For example, after cryopreservation of bull semen, 16 proteins underwent significant changes in abundance [47], while in common carp sperm following cryopreservation, 14 proteins were significantly altered [11]. Similarly, in the present study, using 2D-DIGE coupled with MALDI-TOF/TOF, significant changes in the abundance of 20 proteins were detected in sterlet sperm after cryopreservation. According to the IPA, most of the affected proteins, such as L-lactate dehydrogenase A chain, phosphoglycerate kinase, creatine kinase B-type-like isoform X1, isocitrate dehydrogenase [NAD] subunit alpha, and mitochondrial proteins, are related to various metabolic and energy production processes. Furthermore, some of the proteins, such as the tubulin alpha chain and tubulin beta-4B chain, are part of the cytoskeleton and are involved in spermatozoon motility initiation and maintenance [28]. As a consequence, all of these changes are involved in structural integrity, various biological processes, and the main functions of sperm, ultimately decreasing their fertilization capacity.

The significant decrease in protein abundance or even spot disappearance resulting from the cryopreservation procedure may be due to leakage of proteins from inside the spermatozoa to the extracellular medium [11, 48]. Nevertheless, it has been shown that cryopreservation can also increase the contents of some proteins in spermatozoa through their modification [49, 50]. This suggests that cryopreservation has a complex effect on sperm proteins.

Nevertheless, many previously published results are not as clear, as they are based on analysis of cryopreserved sperm suspensions that contain not only the fraction of interest (viable spermatozoa) but also other constituents generated by the cryopreservation of semen. Usually, under the influence of cryo-damage, to which low-quality spermatozoa could be more susceptible, the membrane-bound proteins and intracellular enzymes, in addition to other components of the spermatozoon, co-elute from spermatozoa into the global sperm suspension. Thus, after analysis of such a suspension, the output results will be quite probably based on the parameters of a mixture of viable, lethal and sublethal damaged spermatozoa subpopulations.

To validate this hypothesis, the analysis of only sperm subpopulations that contain more than 95% of viable spermatozoa selected by Percoll separation was performed in our study. The analysis of this particular sperm fraction showed only one significantly altered protein after the cryopreservation procedure. The identified protein, tubulin alpha-8 chain-like, is linked to cytoskeletal proteins and is involved in sperm motility initiation and maintenance [28]. This result shows a much lower number of changes in proteins from cryopreserved and Percoll separated spermatozoa. However, it is still remains a question if this sperm subpopulation can be more cryoresistant than one that underwent pernicious changes in the proteins. Additionally, the differences in protein abundance when fresh and fresh-separated and cryopreserved and cryopreserved-separated groups were compared are related to the application of Percoll separation. This could result from the fact that the separation technique also removes the proteins that were initially present in the seminal fluid.

Additionally, principal component analysis showed that the greatest changes in protein abundance occurred in the group of sperm samples after cryopreservation compared to the three other groups. The highest differences were observed between fresh sperm and the cryopreserved group, suggesting to what extent cryopreservation can influence the protein profile of spermatozoa, leading to possible protein modification or their leakage to the external medium. However, the changes in protein abundance in Percoll separated samples are present to a much lower extent. These findings suggest that Percoll separation is relevant for both fresh and cryopreserved sperm, as it can remove the low-density spermatozoa and debris present in fresh samples or induced by cryopreservation. Moreover, both fresh and cryopreserved sperm after Percoll separation showed the lowest possible variance in multidimensional space. This indicates that the protein content of cryopreserved Percoll separated samples may not be changed due to cryopreservation and remains the same as in Percoll separated fresh sperm samples. However, the question remains as to whether the separation technique for only selected spermatozoa could be used for the improvement of the fertilization procedure.

In summary, our study, which was performed with four experimental groups of samples, has confirmed that cryopreservation has a deleterious effect on fish sperm motility, viability and the functional state of many sperm proteins in the cryopreserved group when compared to the fresh group. Furthermore, the differences in many proteins found in these groups are related to the presence of lethal and sublethal damaged sperm. Additionally, the differences in protein abundance when the fresh and fresh-separated groups and cryopreserved and cryopreserved-separated groups were compared are related to the application of Percoll separation. This could result from the fact that the separation technique also removes the proteins that were initially present in the seminal fluid.

However, through the application of Percoll separation method to fresh and cryopreserved groups, it is possible to select and analyze only the sperm population that contains more than 95% of viable cells. This selected sperm subpopulation retained high motility parameters before the cryopreservation procedure (fresh-separated group) and after freeze-thawing (cryopreserved-separated group). No differences in sperm motility, viability, or sperm proteins were found in these groups. All of the detected changes in post-thaw sperm motility parameters, viability, and the protein profiles of viable spermatozoa after the freeze-thaw process provide a background for further directions of investigations aimed at a deeper understanding of the mechanism responsible for the cryodamage of sperm. Further studies should also address whether sperm separation techniques can serve as useful tools for both a better understanding of non-lethal sperm damage and the improvement of *in vitro* fertilization results in sturgeon species.

## Supporting information

S1 Fig Visual presentation of results of Percoll gradient centrifugation applied to sterlet sperm. Data on sperm motility in each layer are presented as the mean ± SD, (n = 7).
1st layer: this layer contained mass of spermatozoa and debris; after transferring into activating medium, an extremely low motility percentage (3 ± 1%) was observed;
2nd layer: this layer contained mainly dead spermatozoa and a small amount of debris; after transferring into activating medium, the motility percentage was (18 ± 4%);
Pellet: the pellet contained spermatozoa, debris was not detected; after transferring into activating medium, the motility percentage was 95–100%.
(JPG)

S1 Table Sperm concentration of fresh and cryopreserved samples before and after Percoll separation. Data are presented as median values with the 25% and 75% percentiles (n = 11).
(PDF)
