# Supplementary figures and images for: Cryopreservation effects on a viable sperm sterlet (Acipenser ruthenus) subpopulation obtained by a Percoll density gradient method
Source: PLoS One. 2018 Aug 16;13(8):e0202514. doi: 10.1371/journal.pone.0202514 (PMC6095596; doi:10.1371/journal.pone.0202514)

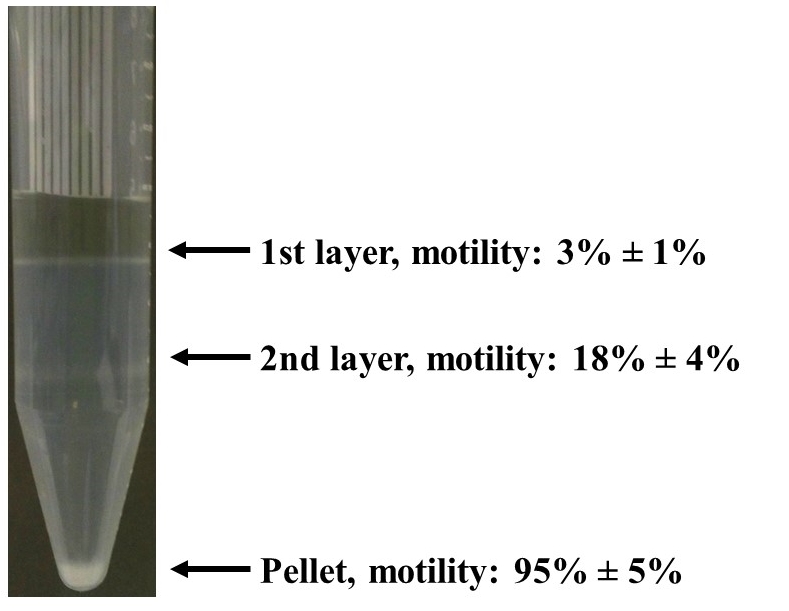

Supplement: S1 Fig — Data on sperm motility in each layer are presented as the mean ± SD, (n = 7). 1st layer: this layer contained mass of spermatozoa and debris; after transferring into activating medium, an extremely low motility percentage (3 ± 1%) was observed; 2nd layer: this layer contained mainly dead spermatozoa and a small amount of debris; after transferring into activating medium, the motility percentage was (18 ± 4%); Pellet: the pellet contained spermatozoa, debris was not detected; after transferring into activating medium, the motility percentage was 95–100%. (JPG) [file pone.0202514.s001.jpg]
