# Supplementary material for: Cryopreservation effects on a viable sperm sterlet (Acipenser ruthenus) subpopulation obtained by a Percoll density gradient method
Source: PLoS One. 2018 Aug 16;13(8):e0202514. doi: 10.1371/journal.pone.0202514 (PMC6095596; doi:10.1371/journal.pone.0202514)
Supplement: S1 Table — Data are presented as median values with the 25% and 75% percentiles (n = 11). (PDF) [file pone.0202514.s002.pdf]

**Parameter**	**Median**	**Percentile 25%**	**Percentile 75%**
Fresh	0.84	0.49	1.24
Fresh-separated	0.68	0.40	1.05
Fresh-separated/Fresh * 100%	80.95	78.84	84.16
Cryopreserved	0.43	0.33	0.86
Cryopreserved-separated	0.13	0.11	0.24
Cryopreserved-separated/Cryopreserved * 100%	28.00	28.00	31.00
